# High-frequency ultrasound processes as alternative methods for degrading meropenem antibiotic in water

**DOI:** 10.1016/j.mex.2022.101835

**Published:** 2022-08-28

**Authors:** Kevin Celis-Llamoca, Efraím A. Serna-Galvis, Ricardo A. Torres-Palma, Jessica I. Nieto-Juárez

**Affiliations:** a Research Group in Environmental Quality and Bioprocesses (GICAB), Faculty of Chemical Engineering and Textile, Universidad Nacional de Ingeniería UNI, Av. Túpac Amaru No 210, Rímac, Lima, Perú; b Grupo de Investigación en Remediación Ambiental y Biocatálisis (GIRAB), Instituto de Química, Facultad de Ciencias Exactas y Naturales, Universidad de Antioquia UdeA, Calle 70 No. 52-21, Medellín, Colombia; c Grupo de Catalizadores y Adsorbentes (CATALAD) Instituto de Química, Facultad de Ciencias Exactas y Naturales, Universidad de Antioquia UdeA, Calle 70 # 52-21, Medellín, Colombia

**Keywords:** Pollutants elimination, Sonochemistry, Water treatment

## Abstract

β-lactam, more specifically carbapenems, are antibiotics used as last resort pharmaceuticals to deal with infections. Despite the medical relevance, they are considered contaminants of emerging concern in water because of their recalcitrance to conventional systems in the municipal wastewater treatment plants. This work aimed to show alternative methods based on the use of high-frequency ultrasound (200-1000 kHz) at a laboratory scale to degrade meropenem (a representative carbapenem antibiotic) in water. The ability of the sonochemical method alone to eliminate meropenem was tested initially. Then, the improvements of degradation by the addition of ferrous iron, or Fe (II) plus UVA light (sono-Fenton, or sono-photo-Fenton methods) were assessed. Finally, the effect of the best ultrasound-based method on the removal of biological activity of meropenem was determined.

• Three high-frequency ultrasound processes were applied to degrade meropenem in water.

• Sono-photo-Fenton degraded 67% of imipenem at 60 min of treatment and decreased significantly H_2_O_2_ accumulation.

• Antimicrobial activity was removed after only 30 min of sono-photo-Fenton action.

## Graphical abstract

**Figure:**
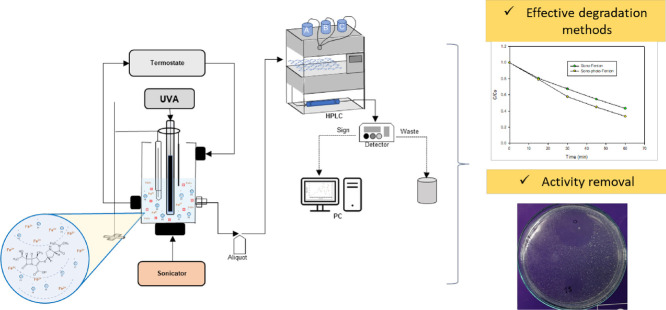

Specifications table

**Table:** 

Subject area:	Environmental Science
More specific subject area:	*Water treatment and organic pollutants degradation*
Name of your method:	*High-frequency ultrasound as an alternative method for degrading meropenem antibiotic in water*
Name and reference of original method:	*D.M. Montoya-Rodríguez, E.A. Serna-Galvis, F. Ferraro, R.A. Torres-Palma, Degradation of the emerging concern pollutant ampicillin in aqueous media by sonochemical advanced oxidation processes - Parameters effect, removal of antimicrobial activity and pollutant treatment in hydrolyzed urine, J. Environ. Manage. 261 (2020).*https://doi.org/10.1016/j.jenvman.2020.110224*.*[1]
Resource availability:	*All resources are detailed within this article*

## Method details

### Introduction

After consumption, antibiotics are excreted/released by the patients into the sewage systems. Then, antibiotics enter the municipal wastewater treatment plants, where typical flocculation/sedimentation and biological processes are unable to eliminate them. Indeed, antibiotics such as meropenem have poor biodegradability and can disturb the wastewater treatment process and the microbial ecology in surface water, even promoting the development/proliferation of antibiotic-resistant bacteria [2].

Due to the limitation of the conventional processes, antibiotics end up in the natural media especially in the aquatic environment [3,4]. Hence, effective treatments to control the input of antibiotics into the aquatic environment are needed. Sonochemical-based processes are alternative methods to eliminate organic pollutants in water, involving the action of hydroxyl radical generated by acoustic cavitation [1]. In these processes, the interaction of high-frequency (200-1000 kHz) ultrasound waves [represented by “)))”], promotes the vapor water and oxygen molecules cleavage Eqs. 1-(4). Moreover, hydrogen peroxide is formed (Eq. 5), which is employed as an indicator of sonochemical activity [1].

(1) H_2_O +))) → ^·^H + ^·^OH

(2) O_2_ +))) → 2 ^·^O

(3) H_2_O + ^·^O → 2 ^·^OH

(4) O_2_ + ^·^H → ^·^O + ^·^OH

(5) 2 ^·^OH → H_2_O_2_

Considering the concerns of antibiotics such as carbapenems and the degrading capability of sonochemical processes, this work aimed to evaluate three high-frequency ultrasound techniques (sonolysis, sono-Fenton, and sono-photo-Fenton) as alternative treatments to eliminate meropenem in aqueous samples.

### Materials, equipment, and analyses

Meropenem trihydrate was purchased from Matrix Scientific, USA. Ammonium heptamolybdate tetrahydrate and sodium acetate trihydrate were obtained from J.T. Baker, Spain. Ammonium chloride, monopotassium phosphate, potassium chloride, calcium chloride, sodium chloride, sodium sulfate, and urea were purchased from Merck Peruana S.A. Iron sulfate heptahydrate and catalase (2000-5000 units mg^−1^) were acquired from Sigma-Aldrich, USA. Hydroxylamine hydrochloride (Thermo Scientific, USA), ortho-phenanthroline (Carlo Erba). Acetonitrile (HPLC grade), citric acid monohydrate; sodium hydroxide (Fisher Chemical), and potassium iodide (Fisher Chemical, USA) were used.

A Meinhardt Ultrasound reactor (containing 300 mL of the antibiotic solution to be treated, at 578 kHz of frequency and 23.8 W of acoustic power) was utilized at a laboratory scale. The temperature of the reactor was controlled at 19 ± 2 °C) using a Brookfield thermostat. A Philips UVA lamp (F4T5BLB) with maximum emission at 365 nm), which was placed on a quartz tube and submerged into the ultrasonic reactor, was utilized for the sono-photo-Fenton method. The acoustic power inside the ultrasound reactor was determined calorimetrically [5]. All experiments were performed at least by duplicate and the average values with their standard deviations were reported.

The meropenem degradation was followed at 300 nm using an HPLC Agilent 1100 equipped with a diode array detector (DAD) and a Teknokroma C-18 column (5 µm, i.d. = 4.6 mm, length = 150 mm). A mixture of acetonitrile/water (25:75 v/v) at 0.4 mL min^−1^ was the mobile phase. The injection volume was 5 µL and the running time was 6 min. Before the chromatographic analyses, catalase (100 µL) was added to the samples to scavenge the residual hydrogen peroxide.

The accumulation of hydrogen peroxide was measured using the iodometric/spectrophotometric method using potassium iodide and ammonium heptamolybdate as detailed in [6].

Antimicrobial activity (AA) against meropenem-sensitive *Staphylococcus aureus* was evaluated by analyzing the inhibition zone in the agar diffusion test following the procedure described in [7].

### Assessment of radicals generation by the ultrasound reactor

**Fig. 1:**
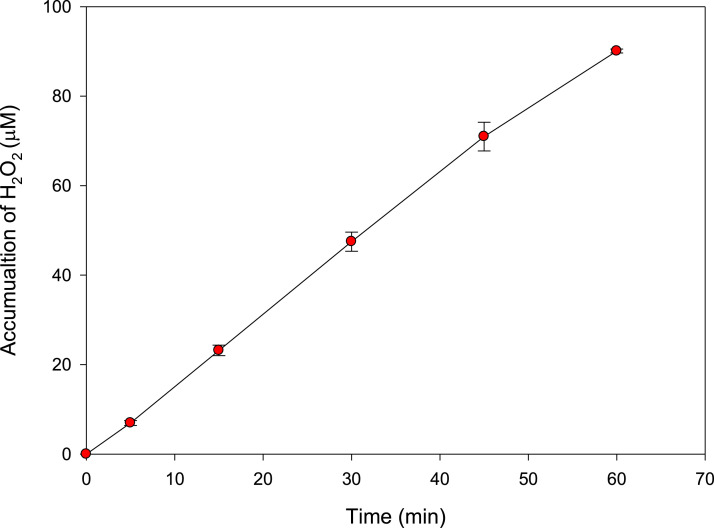
Evolution of H_2_O_2_ accumulated from the sonication of distilled water at 578 kHz and 23.8 W.

To verify the production of hydroxyl radical by the ultrasound reactor, the H_2_O_2_ accumulation during sonication of distilled water at 578 kHz and 23.8 W was determined. Fig. 1 shows that the sonochemical reaction accumulated ∼ 90 µM of hydrogen peroxide after 60 min of sonication, thus confirming the capability of such a reactor to produce radicals such as HO^·^, which in the absences of pollutants fastly evolves to a more stable substance (H_2_O_2_, Eq. 5).

### Degradation of meropenem by sonolysis

**Fig. 2:**
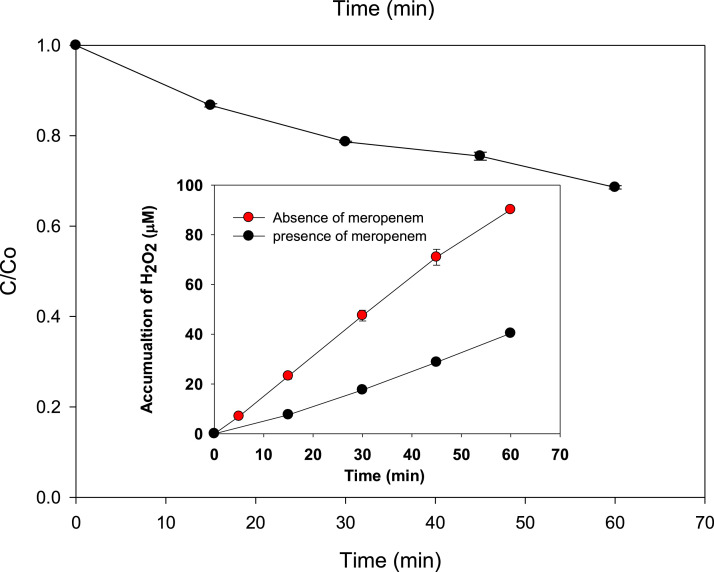
Degradation of meropenem by sonolysis at 578 kHz and 23.8 W. *Inset:* comparison of H_2_O_2_ accumulation in absence and presence of the antibiotic.

The sonochemical method (i.e., sonication at 578 kHz and 23.8 W) was applied to meropenem. The normalized antibiotic concentration progress is shown in Fig. 2. After 60 min of treatment, ∼ 32% of this antibiotic was degraded. Meropenem is not a volatile compound, thus its degradation can be ascribed to the attack of sonogenerated radicals mainly [1]*.* In fact, the inset of Fig. 2 compares the H_2_O_2_ accumulated in the absence and presence of meropenem. Clearly, the hydrogen peroxide accumulation in the pollutant presence was lower than in distilled water alone. This difference supports the interaction of the pharmaceutical with the sonogenerated HO^·^, which leads to meropenem degradation.

### Degradation improvement by ferrous iron and UVA light

**Fig. 3:**
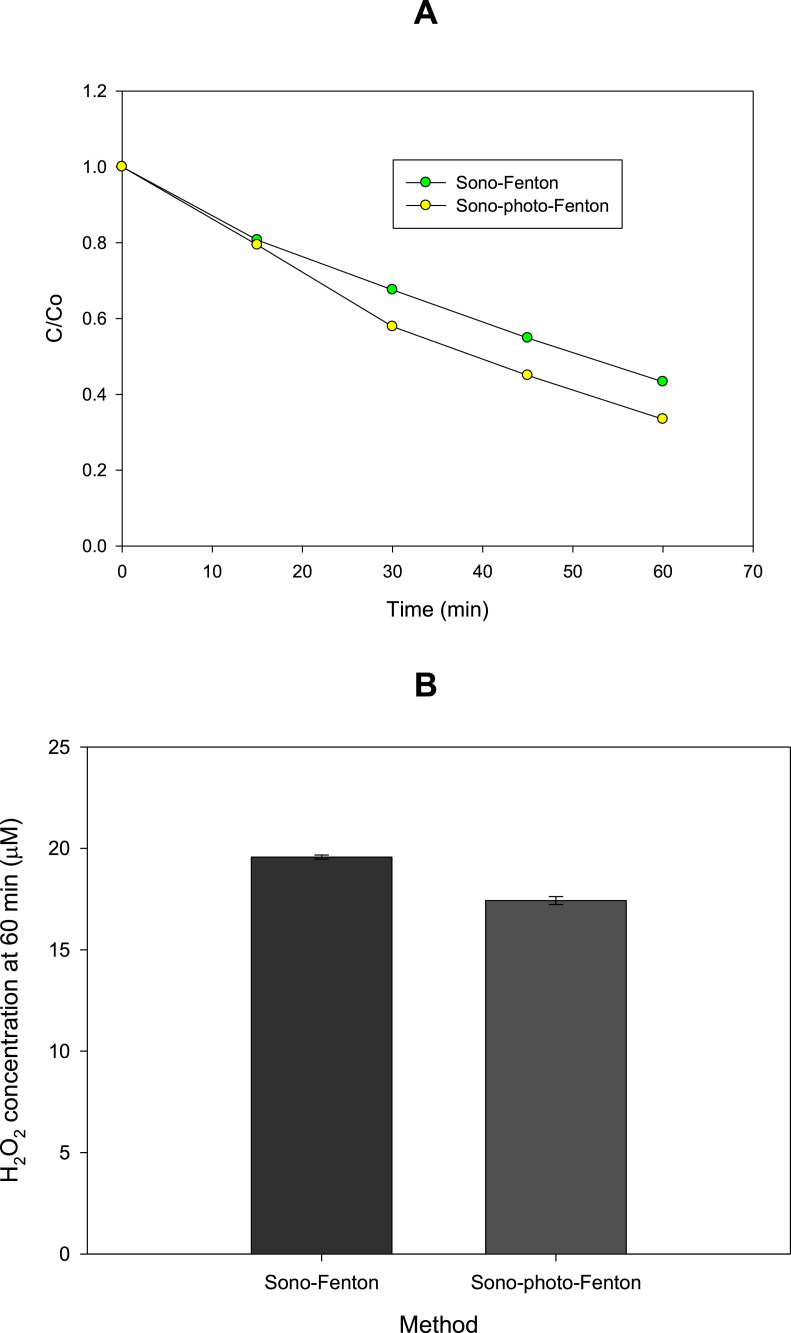
Treatment of meropenem by sono-Fenton and sono-photo-Fenton methods. A. Pollutant degradation. **B.** H_2_O_2_ concentration. Experimental conditions: ultrasound at 578 kHz, 23.8 W, [Fe^2+^]: 5 mg L^−1^, and UVA light (4W), 300 mL.

Ferrous ions at 5 mg L^−1^ (coming from ferrous sulfate heptahydrate) were added to the ultrasound reactor (sono-Fenton method). Also, the effect of simultaneous addition of iron (II) and UVA light (generating the sono-photo-Fenton method) was tested. Then, the evolutions of meropenem, and H_2_O_2_ were followed for 0, 15, 30, 45, and 60 min Fig. 3). It can be noted that, after 1 h of treatment, the sono-Fenton and sono-photo-Fenton methods degraded ∼ 57 and 67% of meropenem, respectively (Fig. 3A). These degradation improvements regarding the sonolysis procedure (which removed ∼ 32 % of meropenem) can be related to Fenton (Eq. 6) and photo-Fenton (Eqs. 6 and (7) reactions that take advantage of the *in situ* sonogenerated H_2_O_2_ to form extra hydroxyl radicals able to attack meropenem [1,8]. This is supported by the low hydrogen peroxide concentration when sono-Fenton and sono-photo-Fenton were applied to degrade the pollutant (see Figs. 2 and 3B).

(6) Fe^2+^ + H_2_O_2_ → Fe^3+^ + HO^·^ +OH^−^

(7) Fe^3+^ + H_2_O + UVA → Fe^2+^ + HO^·^ + HO^−^

On the other hand, we should mention that the sonochemical-based processes effectively led to the degradation of the meropenem (Figs. 2 and 3), which contrasts with the conventional methods used the wastewater treatment systems. For instance, a previous work has shown that meropenem is not biodegradable and this antibiotic can affect bacteria growth, indicating the typical biological process is not able to degrade meropenem [2]. Also, activated carbon is used to remove organic compounds as pharmaceuticals from water. However, this adsorption process moves the pollutant from aqueous media to a solid phase (non-degradation), requiring posterior/extra disposal or treatment of the polluted activated carbon to regenerate it [3]. Chlorination is another classical treatment method for water-containing pharmaceuticals but these pollutants are rich in functional groups very reactive toward chlorine, leading to the formation of chlorinated byproducts, which, in many cases, are highly toxic and carcinogenic [3,9]. Furthermore, many investigations have demonstrated that the use of TiO_2_ as a photocatalyst is effective to eliminate pharmaceuticals and organic compounds in water by the action of hydroxyl radicals. Nevertheless, this system requires that photocatalysts must be removed or recycled at the end of processes, which limits their applications [10].

### Effect of process on the antimicrobial activity

**Fig. 4:**
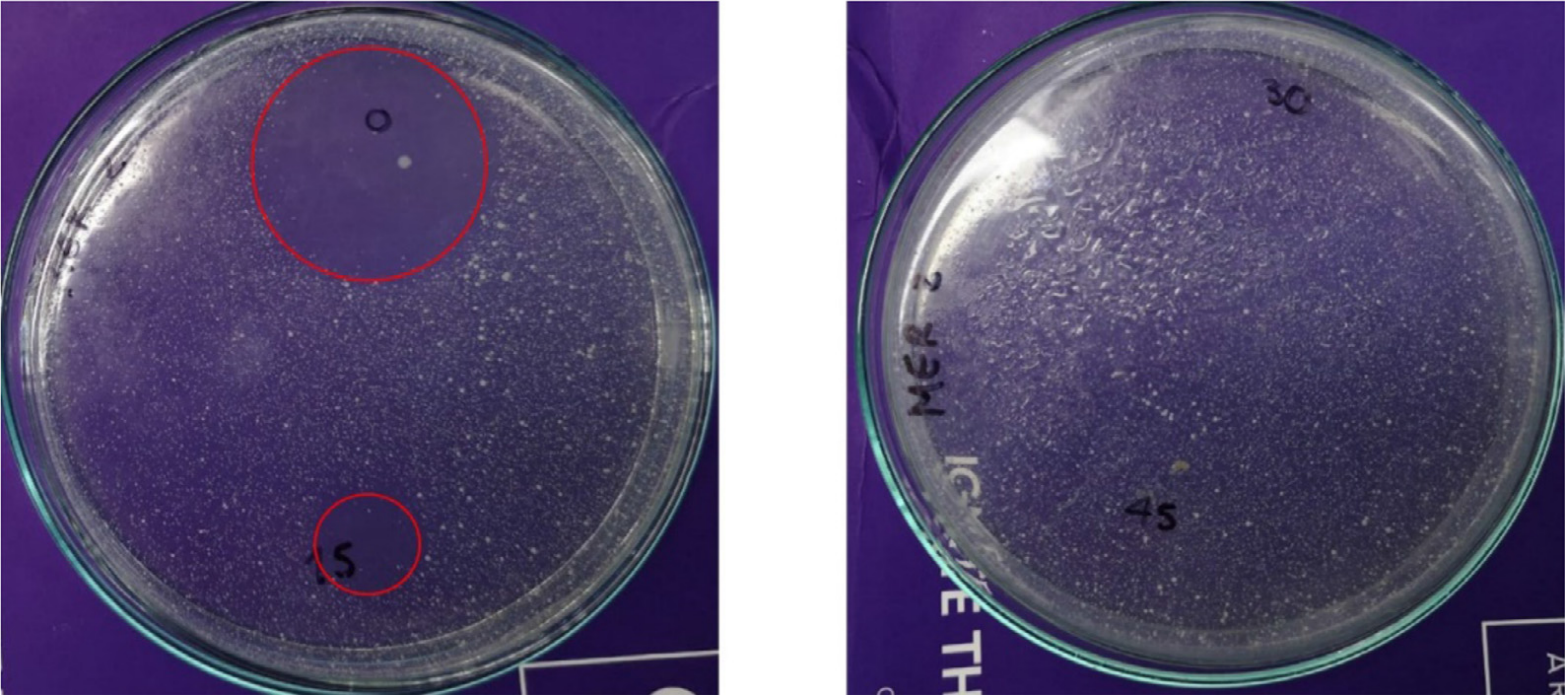
Evolution of antimicrobial activity (AA) against *S. aureus* for meropenem treated by the sono-photo-Fenton method. *Red circles highlight the inhibition zone.*

To demonstrate the action of the sono-photo-Fenton method (which showed elimination capability, Fig 3A), beyond the degradation efficiency, the AA evolution was assessed. Fig 4 presents the inhibitory halo caused by treated meropenem on *S. aureus* at different times (0, 15, 30, and 45 min) of the method application. After 30 min of the treatment with sono-photo-Fenton, there is no inhibitory halo (which corresponded to 43% of meropenem degradation). This indicates that at 30 min of the sono-photo-Fenton action, this process led to imipenem concentration levels below the effective ones for inducing antimicrobial activity [7].

### Final remarks

i) *The high-frequency ultrasound-based processes were able to degrade meropenem in water.*
ii) *Sono-photo-Fenton was the most efficient method to degrade imipenem.*
iii) *Sono-photo-Fenton eliminated the AA after 30 min of treatment even when only 43% of meropenem was degraded.*

## Funding

The authors from GICAB acknowledge the financial support provided by Proyecto de Mejoramiento y Ampliación de los Servicios del Sistema Nacional de Ciencia Tecnología e Innovación Tecnológica 8682-PE, Banco Mundial, CONCYTEC and PROCIENCIA through grant E041-01 (N° 32-2018- FONDECYT-BM-IADT-AV). The authors from GIRAB acknowledge the support provided by Universidad de Antioquia UdeA by means of “Programa de Sostenibilidad”and MINCIENCIAS COLOMBIA through the project No. 1115-852-69594 (PRO-CEC-AGUA). E. A. Serna-Galvis acknowledges MINCIENCIAS COLOMBIA his Ph.D. fellowship during July 2015-June 2019 (Convocation 647/2014).

## CRediT authorship contribution statement

**Kevin Celis-Llamoca:** Investigation, Methodology. **Efraím A. Serna-Galvis:** Investigation, Conceptualization, Methodology, Data curation, Formal analysis, Writing – original draft. **Ricardo A. Torres-Palma:** Conceptualization, Resources, Funding acquisition, Writing – review & editing. **Jessica I. Nieto-Juárez:** Conceptualization, Supervision, Writing – review & editing, Resources, Funding acquisition.

## Declaration of interests

The authors declare that they have no known competing financial interests or personal relationships that could have appeared to influence the work reported in this paper.
